# Some Tributes to the Memory of the Late Dr. Lippe

**Published:** 1888-04

**Authors:** 


### SOME TRIBUTES TO THE MEMORY OF THE LATE DOCTOR LIPPE.

Dr. Bayard :—He was a grand physician. The Ajax of our school, he stood with his broad ax guarding the citadel of truth.

Dr. Hawley :—The news of the death of our venerable colaborer, Dr. Ad. Lippe, was a very great shock to me. We shall miss his ever ready and always ardent defense of our school.

Dr. Ballard :—The loss is not only irreparable, but it is the greatest that could have befallen our school. He has left his handwriting on the wall, where it will be read for all time.

C. B. Gilbert:—Dr. Lippe has been a guide and a counselor ever since I began the study of medicine; knowing his great services to humanity and his firm adherence to true Homœopathy as a physician, I deeply mourn his loss.

Dr. J. A. Biegler:—The announcement of Dr. Lippe's death came to me as an awful visitation which has deeply depressed me. I have known the qualities of character which have made him great among his fellow-workers and revered by all who really knew him. The world has lost a benefactor and we a master mind.

Dr. Kent:—Sad loss, indeed; I loved him deeply. We shall all miss him; he was a master in the art.

Dr. Swan:—I have great respect for his memory as a physician, and consider him to have had no equal in this country and to have been the peer of Bœnninghausen in Europe. And it is a long way down to the next!

Dr. John Hall, Sr.:—The intelligence of his death was a source of deep grief to me, so deep that I hardly know where to go for relief. Many favors and hints have I received at his hands, and these during a long time. Our cause will suffer severely by his loss and all true men will lament him. May his mantle fall on many new and good men and women!

Dr. Julius Schmitt:—His age had prepared all of us to some extent for this great calamity; still, we hoped and prayed that this, our greatest man, might be spared as long as possible. He has left a great many valuable products of his storehouse of homœopathic wisdom; but he could not leave us everything he knew. For me he has been, since I made his acquaintance through his writings, the man to whom I looked up to with the highest reverence and esteem. To master our art as he mastered it has always been my desire, but who can reach that high position from which he looked down on us all?

Dr. Theo. Kafka, Prague:—I beg you to accept my sincerest condolence on that dreadful misfortune you have experienced in the death of your husband, who was one of the stanchest partisans of Homœopathy and one of its greatest savants since Hahnemann.

Dr. Pomeroy:—Whatever others may think or do, it remains true that our school of medicine has but very seldom sustained so great a loss as in the death of Dr. Lippe.

The above are a few extracts from letters received by friends of Dr. Lippe; their publication, we trust, will not be considered a breach of confidence on our part. We also add a few extracts from the papers and journals:

MEDICAL ADVANCE:—* * * * Such is the brief yet sad announcement which terminates the earthly career of perhaps the best-known, the ablest, therapeutist and the most successful prescriber which the American school has produced. A worthy disciple of the immortal Hahnemann both in his teachings and practice, his death leaves a vacancy in our ranks which we fear will long remain unfilled.

MEDICAL COUNSELOR:—The loss of so prominent and valiant a champion of Hahnemannian Homœopathy may be said to fall most heavily on those who share the extreme views ever held and vigorously advocated by the deceased. Yet all believers in homœopathic therapeutics and teaching will sincerely mourn the departure from this life of a man who to the very last displayed a firmness of conviction, a tenacity of purpose, and a degree of consistency which in faithfulness to the "Master," as Lippe ever called Hahnemann, far excelled the example of all other disciples. Indeed, in all the history of Homœopathy not another instance is to be found of a man spending almost half a century in the advocacy of the doctrines of Hahnemann.* * * * If any man ever deliberately consecrated his life to a specific object, then Lippe consecrated his life with all the energy and aggressiveness of his nature to the spread of the doctrines of Hahnemann. The entire and long period of his professional activity was warfare upon each and every one who in any way, shape, or manner transgressed the law of Hahnemann. * * * * Yet it must not be forgotten that he rarely, and perhaps never, entered into a controversy in order to glorify himself. It was always a violation of the teaching of Hahnemann which aroused the ire of the old hero; and the careful reader of the innumerable articles bearing the signature of Ad. Lippe is forced to admit that they are singularly free from egotism. * * * *

No, Lippe belongs to us all. The lessons of his life, in its unmatched consistency and perfect devotion to a conviction, belong to us all. The memory of his perfect surrender of himself to a fixed purpose, of his utter faithfulness at all times and under all circumstances, will be cherished by all. Human imperfections are now forgotten, and the name of Adolph Lippe will not be spoken save in reverence and love.

THE CHIRONIAN:—Professor Adolph Lippe died at his residence, No. 1204 Walnut Street, Philadelphia, on January 23d, after a three days' illness of typhoid-pneumonia.

Homœopathy has always looked up to Professor Lippe as a great teacher, which he truly was in the best sense of the word. His contributions to our knowledge of the materia medica are standard wherever Homœopathy is known, and will be valued the more, now that their talented author has gone.

His reputation spread all over the world and he was recognized as a master.

The loss is a great one; one we know not how to fill.

THE CALIFORNIA HOMŒOPATH:—Adolph Lippe, the old warhorse of pure Homœopathy, died January 23d, 1888, and with him another link is severed which bound the old guard together. Adolph Lippe may have made, by his sturdy ways of detecting fatal errors, many professional opponents; but even they have to acknowledge now that our deceased teacher was not only honest in his convictions, but he dared to offer battle to all who in their mode of constructing Homœopathy yielded to the *suaviter in modo* more than to the *fortiter in re*. No alliance with any society who did not fully carry out the principles of the master, as laid down in the *Organon*, was the rule of his life, and he carried out that rule at the bedside and in the literature of the day, and he was a successful healer. It would be well for Homœopathy if we had more of his stamp, and those who knew him best loved him well, despite his rough manners, which resented every infringement on the domain of pure Homœopathy. Blessed is the man who dares to live up to his convictions during a long and well-spent life; truth knows no half-way station, and Adolph Lippe will always be honored and revered as a true follower of Hahnemann. He is gone to join the departed members of the old guard. Only a few are still allowed to tarry, but may the old and the young physicians of our school always follow such a noble example as the departed gave us, and humanity will be the gainer by it.

S. L.

THE NEW YORK MEDICAL TIMES:—Dr. Lippe was an earnest and consistent Hahnemannian, an able and pungent writer, strong in his friendships and unsparing in his denunciation of what he believed to be fraud and hypocrisy. His clinical reports and his translations from the German, French, and Italian of valuable essays and treatises brought him prominently before the medical public. His belief in Homœopathy was so earnest and so entire that he found no language too strong and no criticism too sharp for those who used what to him was a sacred name to cover what he called a "mongrel practice." Almost the last of the so-called high dilutionists, he was found when the summons of death came with his banner flying and his lance in rest dealing sturdy blows for what he believed to be truth. No one, not even his enemies, of whom he had many, will question the honesty of his convictions or the courage and energy with which he defended them.

THE PHILADELPHIA LEDGER (editorial, January 24th):—Dr. Adolph Lippe, who died yesterday morning of typhoid-pneumonia, was a scientist who preferred to be a Philadelphia physician rather than to wear a title of nobility in his native Germany. His successful career of forty-six years as a practitioner according to the strict tenets of Hahnemann won for him very many friends in the circles where his remarkable cures were made. His insight into nervous diseases alone would have made him famous, even if it had not been accompanied by the experience and highest skill of the general practitioner.

The Women's Homœopathic Hospital Association loses in Dr. Lippe a chivalrous knight ever ready to do battle for its devotion to pure charity and its creed of pure Homœopathy. Dr. Lippe's health has been feeble for some time past, and the swift termination of his fatal illness causes little surprise to those friends who have noted that his spirit, for the past year, was stronger than the flesh that inclosed it. With Dr. Hering, who was considerably his senior, gone, and now Dr. Lippe, the two pioneers who made Pennsylvania and Philadelphia famous by their sagacious medical research, the period may well be considered closed that called Homœopathy a "new school" of medicine. The school has grown old enough to show its considerable divergences, or broadening, as some consider it, upon the medical practice of the two men who stand now as exponents of Homœopathy's "old school." There is room for all opinions in the widening ranks of homœopathic advocates and adherents, and all will unite in doing honor to the great medical genius that Adolph Lippe was.

Dr. Wm. P. Wesselhœft:—No more appropriate tribute can be paid to the memory of Dr. Adolph Lippe than to show his great sagacity in the application of medicines in disease. It was not only his great knowledge of the finer and more subtle indications for remedies, as given in our *Materia Medica* or his judicious examinations of patients, which made him an acknowledged master of our art, but mainly that freer and wider application of our law which elevated him to the sphere of the true artist. His readiness and rapidity in getting at the gist of symptoms, even in the most complicated case, could never be called careless or hasty. It reminded me of the words of an eminent artist, who said : " The chief difficulty with most painters is that they see *too much*, and in seeing too much they get confused with endless detail, which leaves their work without character, and they have little to show for their pains."

He knew the value of our art so well that the commonplaces of every disease were almost instinctively avoided by him, and he never lost time in noting worthless signs, always looking and finding with unusual rapidity the salient points in the case before him. He heeded and lived up to the greatest thought of the master: " The physician's business is only with patients, not with diseases."

The cure of the following case will demonstrate what I mean by a freer and wider application of our law of cure :

I had treated the patient more than eighteen months without improvement, except that his great liability to taking cold had become less.

I copy from my record, taken December, 1881:

G. R., aged forty-five, light brunette, married ten years, general appearance healthy.

For six years has had no discharge of semen during coitus.

Occasionally nocturnal emissions.

Erections usually weak, give out during coitus.

Burning in perineum, worse after going to bed, and when thinking of it.

Drawing pains in testicles, with sensation of weakness of genitals.

Occasionally itching, dry eruptions in crotch and inner upper surface of thighs and anus.

With the sensation of weakness of genitals his eyes feel weak.

Very sensitive to cold and changes of atmosphere.

Takes cold easily, usually affecting nose and throat first with dryness, then with watery catarrh and sneezing, or he has aching pains in different parts of body and limbs, changing location frequently.

Twenty years ago had African fever.

Never had gonorrhœa, syphilis, or other eruptions than those mentioned above.

All other functions normal.

While on a visit to Philadelphia he applied to Dr. Lippe, at my advice.

Dr. Lippe wrote me the following letter :

" I find that your patient had diphtheria about ten years ago and was treated with inappropriate mercurials and gargles by Dr. ——. The character of the attack was that it went from one side to the other and finally back again to the original side. Great weakness, almost paralytic, followed the attack, and he thinks he has never regained his full vigor and usual strength since this illness. His acute colds have always the character of shifting pains and change of location. I have given him a dose of Lac can.$^{cm}$, which may be required to be followed by a dose of Pulsatilla."

Suffice it to say that my patient never needed the suggested dose of Pulsatilla.

In three months after his visit to Philadelphia his wife was pregnant. She has since borne two remarkably healthy children.

As far as we know Lac can. has no sexual weakness. That fact disturbed Dr. Lippe very little in his selection. He looked deeper and found the cause and the remedy. *This is true homœopathic pathology.* All the knowledge in the world of the special pathology of this case could have revealed the remedy to no one. To the homœopathic artist, however, it was revealed, and a man regained his manhood and became the father of two children, after ten years of impotence.

This case, from a pathological point of view, reminds me of one related by Dr. C. Dunham. His patient sickened after the violent suppression of indolent sores by knife and external remedies, and his life was despaired of. The case suited for Lachesis, and Dr. Dunham predicted the reappearance of the eruption, which actually occurred several weeks after the administration of the simillimum. This was another instance of *homœopathic pathology.*

Why did I not discover that my patient had had diphtheria ten years before? All I can answer is that he did not tell me, and that I had not the sagacity to ask! and if I had discovered it I doubt very much if I would have thought of Lac can. for this case. I was far too much impressed with the importance and necessity of eliminating a remedy *for the special weakness for which he had appealed for help.*

This is one of the great mistakes many of us are constantly making, and I hope the publication of this case may be as instructive to others as it has been to me.

